# Serum NMR Profiling Reveals Differential Alterations in the Lipoproteome Induced by Pfizer-BioNTech Vaccine in COVID-19 Recovered Subjects and Naïve Subjects

**DOI:** 10.3389/fmolb.2022.839809

**Published:** 2022-04-05

**Authors:** Veronica Ghini, Laura Maggi, Alessio Mazzoni, Michele Spinicci, Lorenzo Zammarchi, Alessandro Bartoloni, Francesco Annunziato, Paola Turano

**Affiliations:** ^1^ Department of Chemistry, University of Florence, Florence, Italy; ^2^ Magnetic Resonance Center (CERM), University of Florence, Florence, Italy; ^3^ Consorzio Interuniversitario Risonanze Magnetiche di Metallo Proteine (CIRMMP), Florence, Italy; ^4^ Department of Experimental and Clinical Medicine, University of Florence, Florence, Italy; ^5^ Infectious and Tropical Disease Unit, Careggi University Hospital, Florence, Italy; ^6^ Flow Cytometry Diagnostic Center and Immunotherapy, Careggi University Hospital, Florence, Italy

**Keywords:** SARS-CoV-2, vaccine, NMR, metabolomics, lipoproteins

## Abstract

^1^H NMR spectra of sera have been used to define the changes induced by vaccination with Pfizer-BioNTech vaccine (2 shots, 21 days apart) in 10 COVID-19-recovered subjects and 10 COVID-19-naïve subjects at different time points, starting from before vaccination, then weekly until 7 days after second injection, and finally 1 month after the second dose. The data show that vaccination does not induce any significant variation in the metabolome, whereas it causes changes at the level of lipoproteins. The effects are different in the COVID-19-recovered subjects with respect to the naïve subjects, suggesting that a previous infection reduces the vaccine modulation of the lipoproteome composition.

## Introduction

While health systems worldwide race to vaccinate people against SARS-CoV-2, several studies have appeared where the measured levels of antibodies in the blood before vaccination and then after each of the two vaccine doses (Ebinger et al., 2021, 2; Mazzoni et al., 2021, 19). These studies have highlighted different response in COVID-19-recovered or naïve subjects in terms of antibody levels, which is the most relevant information for the design and implementations of efficient mass vaccination campaigns in the context of COVID-19 emergency. One of the main outcomes of such studies in mRNA vaccines indicates that subjects who previously had COVID-19 get a strong immune response from a single dose (Levi et al., 2021; Mazzoni et al., 2021).

^1^H nuclear magnetic resonance (NMR) spectroscopy analysis of biofluids produces profiles that show characteristic responses to changes in physiological status and has been used in a few studies in the past to monitor changes in urinary metabolite levels in mice administered different types of influenza vaccines (Sasaki et al., 2019) or to identify serum markers predictive of adverse reactions against smallpox (McClenathan et al., 2017) as well as metabolic signatures of responses induced by a series of commonly used human vaccines, as reviewed in (Diray-Arce et al., 2020). On the other hand, ^1^H NMR has been also successfully used to monitor changes in metabolites and lipoproteins induced by SARS-CoV-2 infection (Bruzzone et al., 2020; Kimhofer et al., 2020; Ballout et al., 2021; Baranovicova et al., 2021; Bizkarguenaga et al., 2021; Julkunen et al., 2021; Lodge et al., 2021; Masuda et al., 2021; Meoni et al., 2021).

Here, we monitored the time-dependent response to the mRNA Pfizer-BioNTech vaccine in a cohort of 20 healthcare workers, 10 of them had a previous history of COVID-19 and 10 were COVID-19 naïve. All of them received two doses, 21 days apart. The NMR spectra of serum samples collected at six different time points were analyzed to monitor time-dependent intra-individual changes induced by vaccination and to explore possible differences between individual previously infected with COVID-19 and individuals without prior infection. While no significant differences between the two groups exist before vaccination, the first dose is sufficient to induce changes in the lipoproteins levels (but not in metabolites), whose size and nature depends upon absence or presence of previous infection. Differences between the two groups of individuals are maintained along the monitored timeline. The second dose is essentially inconsequential in the group of COVID-19-recovered subjects.

## Material and Methods

### Study Design

**FIGURE 1:**
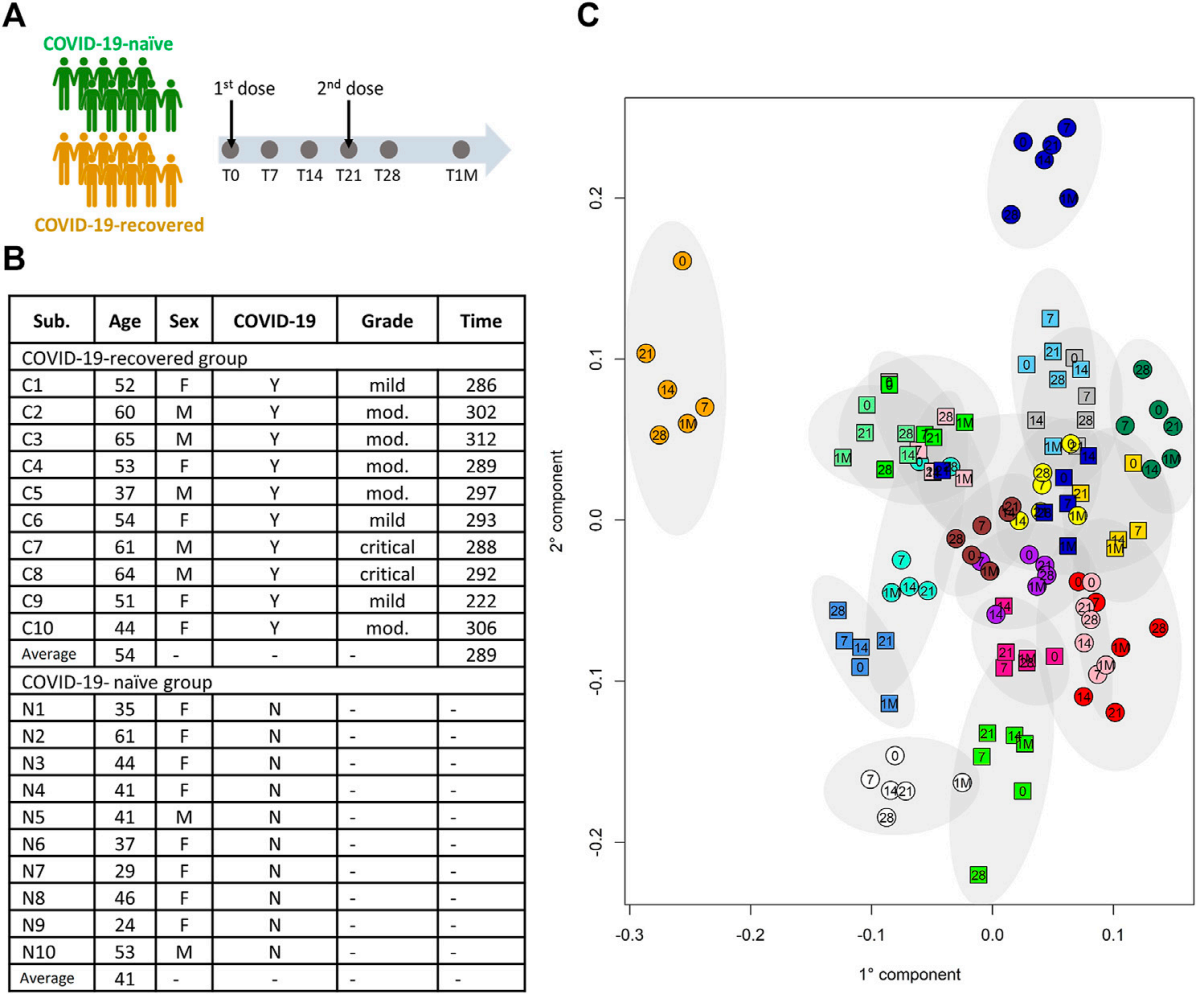
**(A)** Schematic representation of the study design. **(B)** Table summarizing the main demographic characteristics of the subjects included in the study; the COVID-19-recovered and - naïve subjects are indicated with C and N, respectively; for the COVID-19-recovered group the column “Grade” refers to the grade of the disease severity, i.e. mild, moderate (mod.) or critical; the column “Time” refers to the time (in days) from COVID-19 diagnosis to the first dose of vaccine. **(C)** Individual metabolic phenotype as it results from a PCA-CA score plot (binned NOESY spectra). Each color represents a different subject; squares: COVID-19-naïve; circles: COVID-19-recovered. Numbers indicate the collection time: T0 = 0, T7 = 7, T14 = 14, T21 = 21, T28 = 28, T1M = 1M.

The study was conducted at the beginning of the Italian vaccination campaign against COVID-19 using the Pfizer-BioNTech mRNA vaccine (January-February 2021). Twenty Caucasian healthcare workers of the Careggi University Hospital of Florence were recruited, 10 of them had a previous history of COVID-19 (hereafter called “COVID-19-recovered”), and 10 were COVID-19 naïve (“COVID-19-naïve”) (Figure 1A). The main features of the cohort are provided in Figure 1B. The COVID-19-recovered subjects have been infected in the period March-April 2020, with the Wuhan strain; they recovered from the disease on average 255 days before vaccination (range 208–280 days). The inclusion/exclusion criteria were those used for Pfizer-BioNTech vaccine administration for healthcare workers.

The study was conducted in accordance with the Declaration of Helsinki. The study was approved by the Careggi University Hospital Ethical Committee (n. 19466_spe). Written informed consent was obtained from recruited subjects.

For all subjects blood serum samples were collected at six different time points: before the first dose (T0); 7 and 14 days after the first dose (T7 and T14, respectively); 21 days after the first dose, just before the second dose (T21); 28 days after the first dose and 7 days after the second dose (T28); 1 month after the second dose (T1M) (Figure 1A). Blood samples were collected (4 h after breakfast) in a BD vacutainer clot-activator tube for serum collection and processed within 1 hour from sample collection. After processing, all the serum samples were immediately stored at −30°C until NMR analysis (February-March 2021).

### NMR Sample Preparation and Data Acquisition

NMR samples were prepared according to standard procedures (Takis et al., 2019; Vignoli et al., 2019). Frozen serum samples were thawed at room temperature. A total of 350 μl of sodium phosphate buffer (70 mM Na_2_HPO_4_; 20% (v/v) ^2^H_2_O; 6.1 mM NaN_3_, 4.6 mM sodium trimethylsilyl [2,2,3,3−^2^H_4_] propionate (TMSP), pH 7.4) was added to 350 μl of each serum sample; the mixture was homogenized by vortexing for 30 s. A total of 600 μl of each mixture was transferred into a 5.00 mm NMR tube (Bruker BioSpin) for the analysis. ^1^H-NMR spectra were acquired using a Bruker 600 MHz spectrometer (Bruker BioSpin) operating at 600.13 MHz proton Larmor frequency and equipped with a 5 mm PATXI ^1^H−^13^C−^15^N and ^2^H-decoupling probe including a *z* axis gradient coil, an automatic tuning-matching (ATM) and an automatic and refrigerated sample changer (SampleJet, Bruker BioSpin). A BTO 2000 thermocouple served for temperature stabilization at the level of approximately 0.1 K at the sample. Before measurement, samples were kept for 5 min inside the NMR probe head, for temperature equilibration at 310 K.

For each serum sample, three one-dimensional (1D) ^1^H NMR spectra were acquired with water peak suppression and different pulse sequences that allowed the selective observation of different molecular components: 1) a standard NOESY 1Dpresat (noesygppr1d.comp; Bruker BioSpin) pulse sequence (using 32 scans, 98,304 data points, a spectral width of 18,028 Hz, an acquisition time of 2.7 s, a relaxation delay of 4 s and a mixing time of 0.01 s); 2) a standard CPMG (cpmgpr1d.comp; Bruker BioSpin) pulse sequence (using 32 scans, 73,728 data points, a spectral width of 12,019 Hz and a relaxation delay of 4 s); 3) a standard diffusion-edited (ledbgppr2s1d.comp; Bruker BioSpin) pulse sequence (using 32 scans, 98,304 data points, a spectral width of 18,028 Hz and a relaxation delay of 4 s). All spectra were recorded at the Magnetic Resonance Center of the University of Florence (CERM).

Free induction decays were multiplied by an exponential function equivalent to a 0.3 Hz line-broadening factor before applying Fourier transform. Transformed spectra were automatically corrected for phase and baseline distortions and calibrated (glucose doublet at *δ* 5.24 ppm) using TopSpin 3.5 (Bruker BioSpin).

### Assignment and Quantification

The metabolites, whose peaks in the NMR spectra were well defined and resolved, were assigned and their concentrations determined; the assignment procedure was performed using an ^1^H NMR spectra library of pure organic compounds (BBIOREFCODE, Bruker BioSpin). The concentrations of 22 metabolites (Supplementary Table S1) were analysed using *In Vitro* Diagnostics research (IVDr) B.I.-Quant PS tool (Bruker, BioSpin). One hundred fourteen components associated to lipoprotein main parameters, i.e. triglycerides (TG), bound and free cholesterol (Chol and Free Chol), phospholipids (PL), apolipoproteins A1, A2 and B100 (ApoA1, ApoA2 and ApoB100) in each of the main lipoprotein classes, i.e. very low-density lipoproteins (VLDL), high-density lipoproteins (HDL), intermediate-density lipoproteins (IDL), and low-density lipoproteins (LDL) and in their respective subfractions were also analysed (Supplementary Table S2) through the IVDr Lipoprotein Subclass Analysis B.I.-LISA tool (Bruker, BioSpin) (Jiménez et al., 2018).

### Statistical Analysis

All data analyses were performed using the “R” software. Multivariate analyses were applied on NOESY binned spectra. To this aim, each spectrum in the region 10.00–0.2 ppm was divided into 0.02 ppm chemical shift bins, and the corresponding spectral areas were integrated using the AMIX software. The area of each bin was normalized to the total spectral area, calculated with exclusion of the water region (4.50–5.00 ppm). Principal component analysis (PCA) was used as unsupervised exploratory analysis to obtain an overview of the data to detect the presence of clusters (function *prcomp*); canonical analysis (CA) was used in combination with PCA to increase the supervised separation among individuals (in house developed script) and to define their individual metabolomic fingerprint (Assfalg et al., 2008; Bernini et al., 2009). The global accuracy for classification was assessed by means of a Monte Carlo cross-validation scheme.

For univariate analyses, the non-parametric Wilcoxon-Mann-Whitney test was used to infer differences between the metabolite/lipoprotein levels in the comparison between COVID-19-recovered group and COVID-19-naïve group. Instead, for pairwise comparison within each group, the paired Wilcoxon signed-rank test was used to analyzed the differences between the samples of a given individual at each time point with respect to T0 (Neuhäuser, 2011).

## Results

It is known that the NMR detectable part of the blood metabolome/lipoproteome contains a strong signature that defines the individual metabolic phenotype that, in the absence of pathophysiological perturbations, remains stable over a time span of the order of years (Holmes et al., 2008; Yousri et al., 2014; Ghini et al., 2015). The distribution of the metabolic phenotype of the 20 subjects under study is shown in Figure 1C. Notably, we don’t observe any clustering in the metabolic space of the samples from COVID-19-naïve subjects with respect to those of COVID-19-recovered subjects; this result is not unexpected given the fact that COVID-19-recovered subjects are sampled after more than 7 months from infection and do not report any long-COVID symptoms.

**FIGURE 2:**
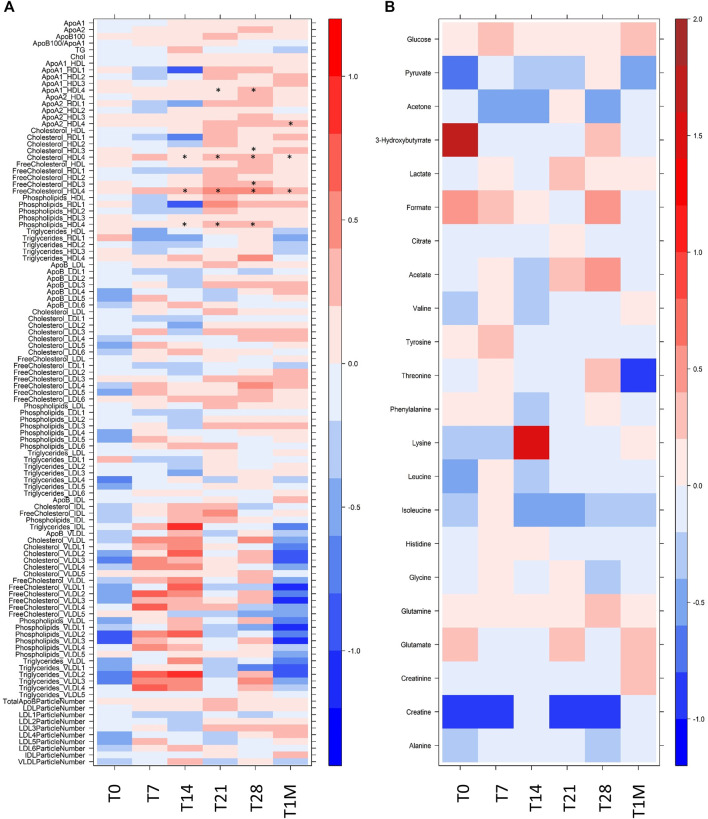
Level plot of Log_2_(FC) of **(A)** lipoprotein related parameters and **(B)** metabolites; red/blue values indicate higher/lower concentration at T0, T7, T14, T21, T28 and T1M samples of COVID-19-recovered group with respect to COVID-19-naïve group. The brightness of each color corresponds to the magnitude of the FC. Asterisks indicate statistical significanceThe level plot has been created using the function *levelplot* implemented in the R package “Lattice”.

As shown in Figure 2, in our cohort the differences that exists at T0 between the two groups are not significant, although the two groups are not identical, as it is normal to expect for the comparison of any 10 randomly selected individuals against any other 10. The intra-individual differences (Figure 2) remain smaller than the inter-individual ones upon vaccination, which therefore does not represent a major modification of the metabolic phenotype. The inter-individual discrimination considering the six samples collected for each subject is >85%. Nevertheless, in response to vaccination we could observe some common changes that are consistently occurring in all subjects within each group at a given time. As shown in Figure 2, the differences between the two groups are essentially restricted to a small number of lipoprotein parameters. They mainly involve HDL4 subfractions (with some *p*-value < 0.05) and appear from T14. Although not statistically significant, a clear trend is observed also for all the VLDL subfractions along the time line T0-T1M; the log_2_(FC) is maximum at T7 and T14 and then decreases, until at T1M it tends towards the re-establishment of the pattern observed at T0.

**FIGURE 3:**
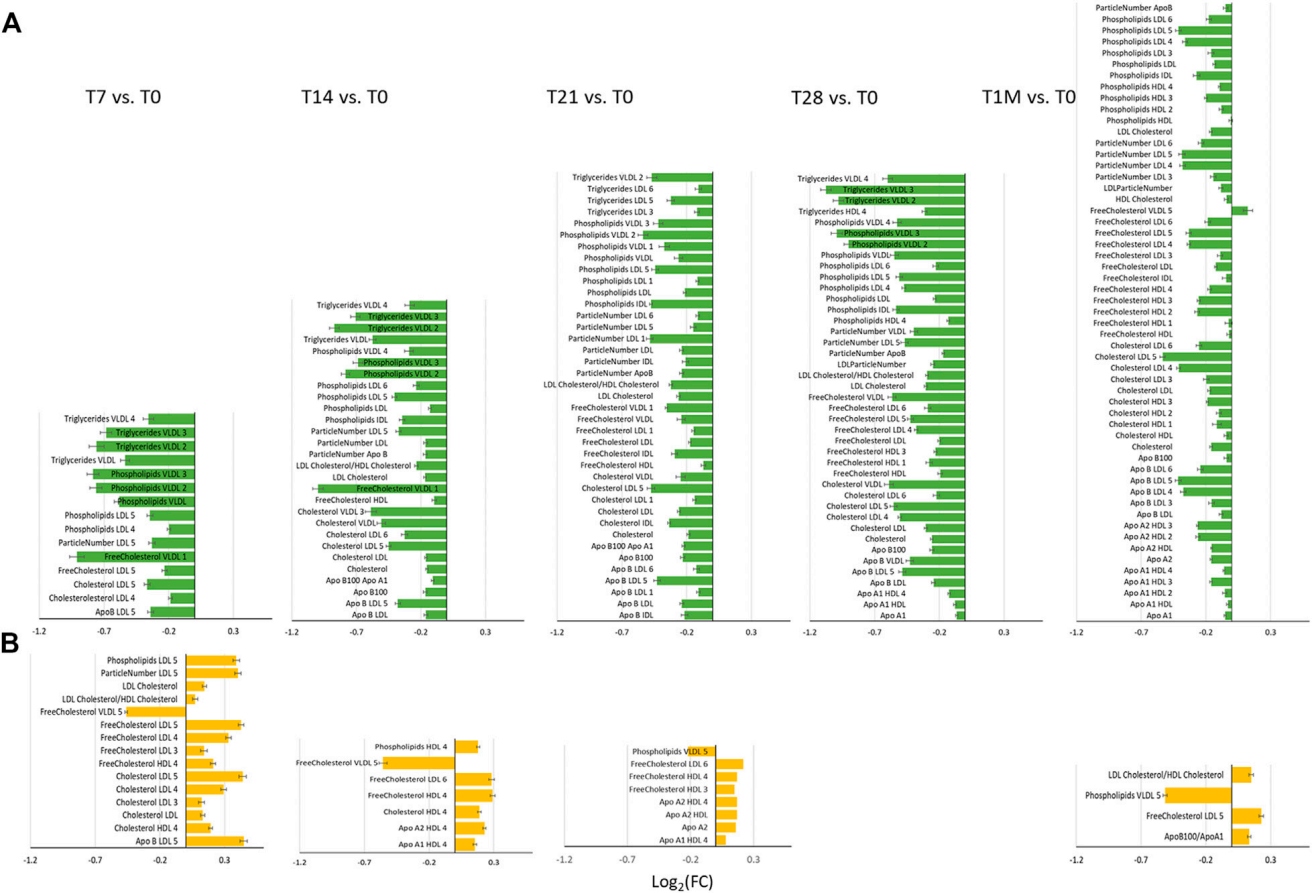
Bar plots of Log_2_ (FC) of lipoprotein related parameters significantly different for the comparison at T7, T14, T21, T28 and T1M with respect to T0, in **(A)** COVID-19-naïve (green plots) and **(B)** COVID-19-recovered (orange plots) groups. Features with Log_2_(FC) positive/negative values have higher/lower concentration in T7, T14, T21, T28 and T1M samples with respect to T0.

**FIGURE 4:**
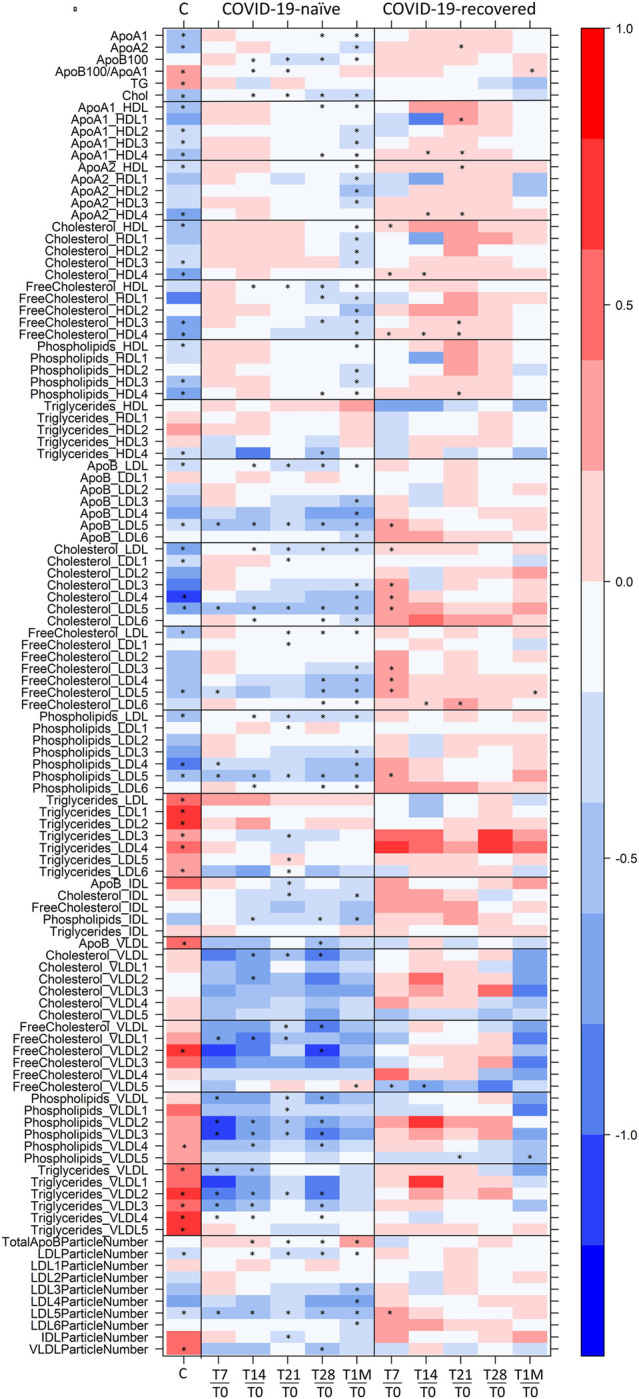
Level plot of Log_2_(FC) of the lipoproteome: for COVID-19-naïve and COVID-19-recovered groups (second and third columns, respectively), red/blue parameters indicate higher/lower concentration at T7, T14, T21, T28, and T1M serum samples with respect to T0 samples. For COVID-19 positive subjects (first column), red/blue parameters indicate higher/lower concentration in serum samples of 30 COVID-19 patients with respect to 30 sex- and age-matched control subjects (Meoni et al., 2021). The brightness of each color corresponds to the magnitude of the FC. Asterisks indicate statistical significance. The level plot was created using the function *levelplot* implemented in the R package “Lattice”.

To better analyze the origin of the time-dependent changes, we performed a paired analysis, so to highlight the common intra-individual variations in each group. To this purpose the concentration of all measurable species for a given individual at each time point was compared to that of the same individual at T0. Figure 3 reports the log_2_(FC) of the lipoprotein parameters that were observed to change significantly in the COVID-19-naïve and COVID-19-recovered groups, separately. The pattern of changes is clearly different between the two cohorts. In the former case (Figure 3A), we observe an overall decrease in concentration of lipoproteins with average absolute values decreasing from T7 to T21, and then increasing again after the second dose (T28) and again decreasing at T1M. Contemporarily, when the time distance from dose administration increases, we observe an increase in the number of dysregulated features. With the help of Figure 4, we can identify the following trends. In terms of main parameters, the most affected along the time series are the ApoB100 and total cholesterol. In terms of main fractions, we observed a continuous dysregulation of the LDL parameters, with the only exception of that associated to triglycerides; these changes persist up to T1M. The earliest (T7) changes are associated to the LDL5 subfraction. For VLDL, the affected main parameters are phospholipids and triglycerides; the largest changes are observed at T7 and T28 (i.e. at the first time point evaluated after the first and second dose, respectively), where the absolute values of their Log_2_(FC) is >0.7; these changes do not persist after T28. The HDL subfractions, with the exception of those associated to triglycerides, change significantly only at T1M, but the extent of the changes is quite small. A completely different trend is observed when looking at the lipoproteins in the COVID-19-recovered subjects (Figure 3B), where the changes are much smaller in size, of the opposite sign (with the only exception of the decrease in Free Cholesterol- and Phospholipids-VLDL5), and essentially negligible after the second dose. Also the number of affected features is very small and substantially limited to HDL4 and LDL5 parameters (Figure 3, Figure 4). In neither case, COVID-19-naïve and recovered groups, the measured levels of lipoproteins exceeded the range of values typical of a population of healthy adults (Jiménez et al., 2018). Interestingly, no consistent changes could be observed for any of the metabolites at any of the sampled time points, in neither group.

## Discussion

^1^H NMR provides a unique tool to measure the levels of lipoprotein main parameters, main fractions and subfractions (Jiménez et al., 2018), in addition to metabolites. Here, NMR allowed us to monitor the effects of the Pfizer-BioNTech vaccine in people who never had a contact with the virus and in those with prior COVID-19 infection. In the former group, changes are relatively large in size and mainly involve a downregulation of LDL -cholesterol, -free cholesterol, –phospholipids and–apolipoprotein B100 along with a downregulation of VLDL-phospholipids and–triglycerides; LDL5 emerges as the main dysregulated subfraction. In the latter group instead, the overall changes are small and limited to few lipoprotein components (HDL4 and LDL5 features).

Although this is a small-size pilot study, those described above are clear-cut differences that is extremely unlikely to happen due to chance. The interpretation of the observed changes is far from straightforward. An obvious comparison is with the immunological response. Indeed, the same subjects have been analyzed by some of us in terms of their immune response (Mazzoni et al., 2021). The anti–SARS-CoV-2 serum antibody levels in COVID-19–recovered subjects reach a plateau after the first dose (T7-T14), without any additional improvement after the second one. Instead, in the COVID-19-naïve subjects these levels are not reached even after the second dose (T28).

There is not a common pattern in the timeline trend of immune response and lipoprotein alterations, the only common trait being a reduced response to the second dose in the COVID-19-recovered subjects. What we observe by NMR is most probably an interplay of multiple effects, with a different modulation in the two groups of vaccinated subjects. The fact that previous infection limits the extent of the observed effects suggests that whatever process remodulates the lipoproteins following vaccination in COVID-19-recovered subjects, it has to be related to the “new” encounter with the spike protein. It is worth noting that lipid stripping from cell membrane is a phenomenon associated to the specific action of the spike protein and might be differently operative in recovered and naïve individuals. It is also known that LDL and cholesterol are key mediators of inflammation (Chróinín et al., 2014), which could also have a different extent in recovered and naïve subjects following vaccination. Notably during acute COVID-19 infection, where both lipid bilayer degradation induced by the spike protein and severe inflammation occur, cholesterol and LDL5 are also significantly altered with respect to healthy values, Figure 4, first column (Bruzzone et al., 2020; Kimhofer et al., 2020; Ballout et al., 2021; Bizkarguenaga et al., 2021; Lodge et al., 2021; Masuda et al., 2021; Meoni et al., 2021).

Although aware of the intrinsic limitations of the study, we believe the results could stimulate future research addressing a number of relevant aspects. This type of results, if confirmed in larger and diverse (by age, sex, ethnicity, morbidities) populations, might help defining abnormal response to vaccination with the Pfizer-BioNTech formulation and adverse events. A comparison between the effects induced by the different vaccines (Pfizer vs. Moderna; mRNA vs. DNA vaccines, etc.) might shed light on the existence of correlations between fluctuations in the lipoprotein profiles and immune status and to dissect them from the response to the specific formulation.

## Data Availability

The dataset presented in this study can be found in an online repository. The name of the repository and accession number can be found below: This study is available at the NIH Common Fund's National Metabolomics Data Repository (NMDR) website, the Metabolomics Workbench, https://www.metabolomicsworkbench.org where it has been assigned Study ID ST002086. The data can be accessed directly *via* its Project DOI: http://dx.doi.org/10.21228/M8FM6D.
