# Supplementary material for: Serum NMR Profiling Reveals Differential Alterations in the Lipoproteome Induced by Pfizer-BioNTech Vaccine in COVID-19 Recovered Subjects and Naïve Subjects
Source: Front Mol Biosci. 2022 Apr 5;9:839809. doi: 10.3389/fmolb.2022.839809 (PMC9037139; doi:10.3389/fmolb.2022.839809)
Supplement: Supplementary file 1 [file DataSheet1.docx]

| **Metabolites** |
| --- |
| 3-Hydroxybutyrrate |
| Acetate |
| Acetone |
| Alanine |
| Citrate |
| Creatine |
| Creatinine |
| Formate |
| Glucose |
| Glutamate |
| Glutamine |
| Glycine |
| Histidine |
| Isoleucine |
| Lactate |
| Leucine |
| Lysine |
| Phenylalanine |
| Pyruvate |
| Threonine |
| Tyrosine |
| Valine |

**Table S1**. List of analysed metabolites

| **Lipoproteins** |
| --- |
| **Main Parameters** |
| Triglycerides (TG) |
| Total Cholesterol (Cholesterol) |
| LDL- Cholesterol |
| HDL- Cholesterol |
| Apolipoprotein A1 (ApoA1) |
| Apolipoprotein A2 (ApoA2) |
| Apolipoprotein B100 (ApoB100) |
| **Calculated ratio** |
| ApoB100/ApoA1 |
| HDL Cholesterol/LDL Cholesterol |
| **Total concentration of ApoB carrying particles** |
| Total ApoB particle number |
| Lipoprotein main Fractions |
| VLDL Particle Number |
| IDL Particle Number |
| LDL Particle Number |
| LDL subfractions |
| LDL1 Particle Number |
| LDL2 Particle Number |
| LDL3 Particle Number |
| LDL4 Particle Number |
| LDL5 Particle Number |
| LDL6 Particle Number |
| **Lipoprotein main fraction composition** |
| Triglycerides |
| Triglycerides-VLDL |
| Triglycerides-IDL |
| Triglycerides-LDL |
| Triglycerides-HDL |
| Cholesterol |
| Cholesterol-VLDL |
| Cholesterol-IDL |
| Cholesterol-LDL |
| Cholesterol-HDL |
| Free cholesterol |
| Free Cholesterol-VLDL |
| Free Cholesterol-IDL |
| Free Cholesterol-LDL |
| Free Cholesterol-HDL |
| Phospholipids |
| Phospholipids-VLDL |
| Phospholipids-IDL |
| Phospholipids-LDL |
| Phospholipids-HDL |
| ApoA1 |
| ApoA1-HDL |
| ApoA2 |
| ApoA2-HDL |
| ApoB |
| ApoB-VLDL |
| ApoB-IDL |
| ApoB-LDL |
| **Lipoprotein subfraction composition** |
| **VLDL subfractions** |
| Triglycerides |
| Triglycerides-VLDL1 |
| Triglycerides-VLDL2 |
| Triglycerides-VLDL3 |
| Triglycerides-VLDL4 |
| Triglycerides-VLDL5 |
| Cholesterol |
| Cholesterol-VLDL1 |
| Cholesterol-VLDL2 |
| Cholesterol-VLDL3 |
| Cholesterol-VLDL4 |
| Cholesterol-VLDL5 |
| Free cholesterol |
| Free Cholesterol-VLDL1 |
| Free Cholesterol-VLDL2 |
| Free Cholesterol-VLDL3 |
| Free Cholesterol-VLDL4 |
| Free Cholesterol-VLDL5 |
| Phospholipids |
| Phospholipids-VLDL1 |
| Phospholipids-VLDL2 |
| Phospholipids-VLDL3 |
| Phospholipids-VLDL4 |
| Phospholipids-VLDL5 |
| **LDL subfractions** |
| Triglycerides |
| Triglycerides-LDL1 |
| Triglycerides-LDL2 |
| Triglycerides-LDL3 |
| Triglycerides-LDL4 |
| Triglycerides-LDL5 |
| Triglycerides-LDL6 |
| Cholesterol |
| Cholesterol-LDL1 |
| Cholesterol-LDL2 |
| Cholesterol-LDL3 |
| Cholesterol-LDL4 |
| Cholesterol-LDL5 |
| Cholesterol-LDL6 |
| Free cholesterol |
| FreeCholesterol-LDL1 |
| FreeCholesterol-LDL2 |
| FreeCholesterol-LDL3 |
| FreeCholesterol-LDL4 |
| FreeCholesterol-LDL5 |
| FreeCholesterol-LDL6 |
| Phospholipids |
| Phospholipids-LDL1 |
| Phospholipids-LDL2 |
| Phospholipids-LDL3 |
| Phospholipids-LDL4 |
| Phospholipids-LDL5 |
| Phospholipids-LDL6 |
| Apo-B |
| ApoB-LDL1 |
| ApoB-LDL2 |
| ApoB-LDL3 |
| ApoB-LDL4 |
| ApoB-LDL5 |
| ApoB-LDL6 |
| **HDL subfractions** |
| Triglycerides |
| Triglycerides-HDL1 |
| Triglycerides-HDL2 |
| Triglycerides-HDL3 |
| Triglycerides-HDL4 |
| Cholesterol |
| Cholesterol-HDL1 |
| Cholesterol-HDL2 |
| Cholesterol-HDL3 |
| Cholesterol-HDL4 |
| Free cholesterol |
| FreeCholesterol-HDL1 |
| FreeCholesterol-HDL2 |
| FreeCholesterol-HDL3 |
| FreeCholesterol-HDL4 |
| Phospholipids |
| Phospholipids-HDL1 |
| Phospholipids-HDL2 |
| Phospholipids-HDL3 |
| Phospholipids-HDL4 |
| Apo-A1 |
| ApoA1-HDL1 |
| ApoA1-HDL2 |
| ApoA1-HDL3 |
| ApoA1-HDL4 |
| Apo-A2 |
| ApoA2-HDL1 |
| ApoA2-HDL2 |
| ApoA2-HDL3 |
| ApoA2-HDL4 |

**Table S2**. List of analysed lipoprotein main parameters, main fractions and subfractions.
